# Age-related differences in implicit temporal preparation

**DOI:** 10.3389/fpsyg.2026.1734451

**Published:** 2026-03-09

**Authors:** Matthew S. Welhaf

**Affiliations:** Department of Psychology, North Carolina A&T State University, Greensboro, NC, United States

**Keywords:** age-related differences, foreperiod effect, reaction time, sequential effects, temporal preparation

## Abstract

Healthy aging is commonly associated with cognitive changes in several domains. One process that has gained recent attention in the context of cognitive aging is temporal processing of information and preparation for action. The current study aimed to extend recent findings showing that implicit timing ability, or the ability to process temporal information and act when not instructed to attend to time, might be spared in older adulthood. Across two independent studies, younger and older adults completed a simple reaction time task in which a variable foreperiod duration was presented on every trial. I analyzed two aspects of temporal preparation ability, the more controlled “variable” foreperiod effect and the more automatic “sequential” foreperiod effect to see if either of these aspects might be affected by age. In both studies, older adults demonstrated consistent slowing of responses. In terms of indices of temporal ability, however, both younger and older adults showed similar patterns. Older adults showed similar levels of their variable and sequential foreperiod effects compared to younger adults. These findings suggest that implicit timing ability appears relatively unaffected by healthy aging and add to the growing body of literature to better understand foreperiod effects and response timing more broadly.

## Introduction

Our ability to process time and information on the moment-to-moment basis is central to many everyday activities including driving and holding conversations. One was to assess temporal processing ability in this time domain, at least partially, is with “implicit” timing tasks. These tasks are often simple reaction time (RT) task in which there is an unknown duration (or foreperiod; FP) between trial onset (or warning signal) and the occurrence of the target stimulus. In the variable FP paradigm, where the FP varies on each trial within a block, creates maximal temporal uncertainty across the block (Niemi and Näätänen, 1981). Here, RTs tend to decrease as FP duration increases and has been deemed the *variable FP effect* (e.g., Drazin, 1961; Klemmer, 1956; Niemi and Näätänen, 1981; Mattes and Ulrich, 1997). The size of the variable FP effect, then, has been proposed to index the degree of implicit timing ability (e.g., Coull and Nobre, 2008). A second common finding in these implicit timing paradigms is the so-called sequential FP effect. The sequential FP effect arises as RTs on a current trial with a short FP are substantially slowed when they follow a previous trial with a longer FP compared to one that is equally long or shorter (e.g., Karlin, 1959; Los et al., 2001; Steinborn et al., 2008).

There is an ongoing debate in the literature on the variable FP effect and sequential FP effect as to what the underlying mechanisms driving these processes are. Although beyond the scope of this article, I briefly summarize the two main competing views. One prominent account of the variable FP effect is that people rely on information gained during the FP about the probability of an upcoming stimulus. This is represented by a “hazard function,” in which the probability of an event increases as time passes. More specifically, when FP are uniformly distributed in a specific range (i.e., they are equally likely to occur), trials with longer FPs are more quickly responded to because the conditional probability that the trial will occur is greater. This account suggests that people will strategically adjust their behavior by using relevant temporal information even if they are not explicitly told to pay attention to time (e.g., Alegria, 1975; Niemi and Näätänen, 1981). Thus, people are faster to respond at longer duration FPs relative to shorter duration FPs because they can prepare to respond once they realize that shorter FPs are no longer likely to appear on that trial. This “hazard-based” approach also suggests that the variable FP effect occurs in part because of the sequential FP effect (e.g., Los et al., 2001; Los and van den Heuvel, 2001; Los and Heslenfeld, 2005; Niemi and Näätänen, 1981). However, more recent models of temporal preparation take a middle ground stance (e.g., Multiple Trace Theory; Los et al., 2014) and suggest that both the variable and sequential FP effects are affected by different aspects of memory traces. Specifically, the strength of memory traces appears to explain the sequential FP effect, while the number of memory traces can account for the variable FP effect (see Han and Proctor, 2022; Los et al., 2017, 2021; Mattiesing et al., 2017; Salet et al., 2022).

There has been a growth of interest in understanding implicit timing ability in the context of healthy (e.g., Bherer and Belleville, 2004a,b; Chauvin et al., 2016; Droit-Volet et al., 2019; Gallego Hiroyasu and Yotsumoto, 2020; Otsuka et al., 2023; Vallesi et al., 2009; Visalli et al., 2025) and pathological aging (e.g., Capizzi et al., 2022; Jurkowski et al., 2005; Mioni et al., 2018). However, many of these studies focus almost exclusively on the effects of age on the variable FP effect or at most consider the sequential FP effect as a confound (e.g., Visalli et al., 2025). However, both processes may provide important information on changes in implicit timing ability that occur with age.

It is well-established that there are age-related changes in more global cognitive processes such as general processing speed (e.g., Salthouse, 1996) and some attentional control abilities (e.g., Verhaeghen and Cerella, 2002). These age-related changes might also contribute to more specific modulations of temporal preparation ability. However, simple RT tasks with rather limited FP durations (which are commonly used in the FP literature) may not place the same executive and processing speed demands on younger and older adults. As such, using tasks that might expand on these demands can better inform how implicit timing ability might (or might not differ) between younger and older adults. The aim of the current study was to add to the growing body of literature on possible age-related differences in implicit timing ability and to test for both variable and sequential FP effects in younger and older adults in a task that has been known to place higher demands on processing speed and attentional abilities, and age-differences therein.

### Age-related differences in implicit timing

In the context of age-related changes in variable FP effect, several studies have shown that older adults show a larger variable FP effect compared to younger adults (e.g., Bherer and Belleville, 2004a,b; Capizzi et al., 2022; Droit-Volet et al., 2019; but see Vallesi et al., 2009 for evidence of older adults showing a reversed variable FP effect). These findings of larger variable FP effects in older adults have led some to suggest that older adults might rely more on this hazard function than younger adults. However, it is important to note that several studies have also reported similar magnitudes of variable FP effect between younger and older adults (Chauvin et al., 2016; Droit-Volet et al., 2019; Gallego Hiroyasu and Yotsumoto, 2020; Otsuka et al., 2023; Visalli et al., 2025). Thus, it is possible that implicit timing abilities are largely comparable across younger and older adults, and the use of the hazard function is relatively stable between younger and older adults.

To my knowledge, only one study has reported on possible age-related differences in the sequential FP effect (Vallesi et al., 2009). In this study, participants (*n* = 14 young and *n* = 14 old) completed a standard variable FP task. While there was evidence for age-differences in the variable FP effect, Vallesi et al. (2009) found no evidence of age differences in the magnitude of the sequential FP effect. This finding seems to support dual-process models of implicit timing (e.g., Vallesi, 2010) that the variable and sequential FP effects might reflect different mechanisms that are (un)affected by age. Given the dearth of research concerning age-related differences in sequential FP effects, more work is needed to better understand how older adults are affected by previous FPs and where this impact my arise.

## Current study

The main goal of the current study was to add to the growing body of literature on age differences in implicit timing ability as assessed by both the variable and sequential FP effects. To test this, I report data from two samples (one of which reanalyzed data from a previously published study; Maybrier et al., 2023). In each study, participants completed a version of a Psychomotor Vigilance Task (PVT; Dinges and Powell, 1985) which has recently been used to demonstrate FP effects (Massar and Chee, 2015; Massar et al., 2018, 2020; Welhaf, 2024; Yamashita et al., 2022). I hypothesized that if older adults and younger adults differ in their ability to use the hazard function, then I would expect to find differences in the magnitude of the variable FP effect (e.g., Bherer and Belleville, 2004a,b; Droit-Volet et al., 2019). Specifically, older adults would show a larger variable FP effect.

Regarding age-differences in the sequential FP effect, I had no strong hypothesis regarding how age would be related to the magnitude of the sequential FP effect. Instead, I sought to provide any evidence for a possible association.

### Transparency and openness

The sample size justification and data exclusion criteria, as well as all measures and manipulations included in the study are reported below (Simmons et al., 2012). All deidentified data and the Rmarkdown files for analyses are available on the Open Science Framework^1^. Analyses for these two studies were not preregistered. All data aggregation and analyses were performed in R (R Core Team, 2021) using *tidyverse* (Wickham et al., 2019). Data were analyzed using mixed effect models with the *lme4* package. *P*-values were calculated using the default Satterwaithe approximation in the *lmerTest* package. Data visualizations were created using *ggplot2* (Wickham, 2016).

## Study 1

### Participants

Data for this reanalysis were reported by Maybrier et al. (2023). Eighty participants were initially recruited for this study. Younger adults (*n* = 42) were recruited from the Washington University Psychological and Brain Sciences undergraduate subject pool or local St. Louis area via a research recruitment registry. Older adults (*n* = 38) were recruited from the local St. Louis area via the same research recruitment registry. One older adult was excluded due to reporting use of sleep medication.

### Materials and procedures

Participants completed two lab visits approximately 1 week apart. The first session focused on training participants on wearing an actigraphy watch and how to complete a sleep diary. At the start of the second session, they completed the psychomotor vigilance task among other cognitive tasks (e.g., spatial navigation) and several self-report measures. The estimated duration of the second session was roughly an hour.

### Task

#### Psychomotor vigilance task (PVT)

Participants were told to fixate on a crosshair in the center of the screen and respond as quickly as possible when they noticed a red dot appear. The ISI for the appearance of the red dot varied and was unpredictable to participants and ranged from 1000 to 10,000 ms in a continuous distribution. The PVT was programmed in Psychology Experiment Building Language (Mueller and Piper, 2014). Participants completed an average of 81 trials (SD = 15). As in previous work (Welhaf, 2024), I categorized trials as Short or Long FP if they were below or above 6,000 ms. Specifically, trials with a FP between 1,000 and 5,999 ms were considered “short” and trials with an FP ≥ 6,000 ms were considered “long.” This resulted in an average of 46 (SD = 10) trials being “short” and 36 (SD = 8) trials being “long.”

## Results

Consistent with previous work (e.g., Welhaf, 2024), I removed RTs to false alarms (i.e., hitting the spacebar during the ISI) and RTs below 200 ms. Next, RTs greater than 3,000 ms were removed. This resulted in a total of 69 trials being removed across all subjects (1.1% of all trials). Finally, I removed RTs that were clearly not part of participants’ distribution by censoring outlying RTs to a value equal to each individual participant’s median RT + 3⋅IQR (interquartile range). This affected, on average, 2.6% of RTs across all participants.

Additionally, I removed two participants, both older adults, from the original data set as they had less that five trials recorded and thus insufficient information to conduct these analyses. Finally, consistent with previous research, I removed data from one participant who had an excessive number of missing trials as described below (Robison et al., 2023; Welhaf et al., 2025). Thus, the final analyzed sample consisted of 77 participants. Specifically, there were 40 younger adults (27 female) who were, on average, 19.94 years old (SD = 1.51 years) and 37 older adults (17 female) who were, on average, 70.26 years old (SD = 6.92 years).

### Effects of age on implicit timing ability

**FIGURE 1:**
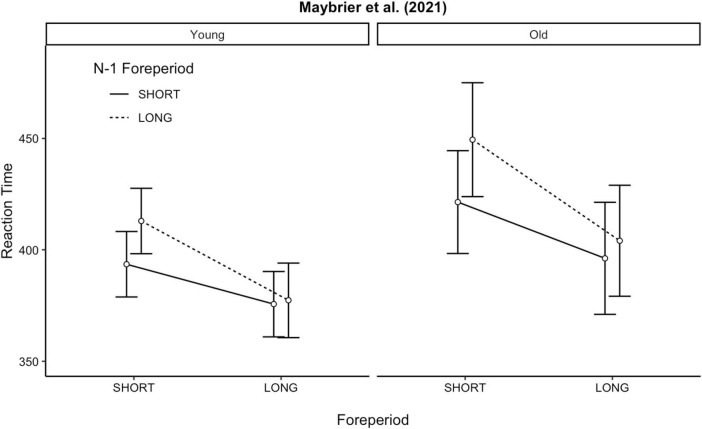
FP effects for each age group. Dots represent the condition mean and error bars are the 95% confidence intervals. FP, foreperiod.

The results of the linear mixed effect model indicated several significant effects, and the results are visualized in Figure 1. There was no significant effect of age on RT, *b* = −22.59 (SE = 14.49), *p* = 0.081. Younger adults (*M* = 389, SD = 51) were only numerically faster to respond during the PVT compared to older adults (*M* = 418, SD = 79).

There was a significant effect of current FP, *b* = 44.55 (SE = 4.36), *p* < 0.001. Across all participants, there was a significant slowing in responses when the current trial was a short FP duration (*M* = 418, SD = 65) compared to when the current trial was a long FP duration (*M* = 387, SD = 65). Thus, there was a robust variable FP effect.

Surprisingly, there was no significant sequential FP effect, *b* = −7.08 (SE = 4.36), *p* = 0.104, but there was a significant asymmetry in the sequential FP effect, *b* = −19.51 (SE = −5.74), *p* < 0.001. Central to the current paper, none of the FP-related effects were moderated by age (*p*’s > 0.084).

### Study 1 discussion

The goal of Study 1 was to investigate implicit temporal preparation abilities in a group of younger and older adults. I hypothesized, consistent with previous work, that there would be a sparing of implicit temporal preparation ability with age. The data from Study 1 supported this hypothesis. While there was robust evidence of the variable FP effect, this was not moderated by age. Additionally, there was no evidence of a sequential FP effect nor an interaction with age. These findings are largely consistent with Visalli et al. (2025) and suggest that implicit processing of time is largely preserved with age.

## Study 2

The findings from Study 1 arose in the PVT which has only recently been shown to demonstrate FP effects in younger adults (e.g., Welhaf, 2024; Yamashita et al., 2022). Thus, it is important to replicate these patterns of spared implicit temporal preparation ability in older adults. Thus, the goal of Study 2 was to replicate the findings of Study 1 in the PVT to address consistencies across paradigms and in a larger sample to increase power to detect any possible age-related differences in temporal preparation.

## Methods

### Participants

We aimed to collect data from 75 younger adults (age range = 18–35 years old) and 75 older adults (age range = 60+ years old) via Prolific Academic^2^ to complete a 15-min study for $3.50. We used G*Power 3.1 to conduct a sensitivity analysis for conducting a repeated measures ANOVA with a within-between interaction. With a sample size of 75 in each group, alpha = 0.05, and power = 0.80, we would be able to detect effects between *f* = 0.103 and 0.115 (based on the FP and sequential FP effect, respectively). To account for possible exclusions and data loss, data from a total of 160 participants was collected (*n*_*older*_ = 80; *n*_*younger*_ = 80).

The study was approved by the Washington University in St. Louis Institutional Review Board. All tasks and questionnaires were programmed in Gorilla^3^ and we required participants to complete the study on a laptop or desktop computer to ensure accurate recording of RTs (Anwyl-Irvine et al., 2020).

### Tasks

#### PVT

Participants completed a PVT designed for online administration (Welhaf and Kane, 2024). On each trial of this task, participants saw a set of zeros (00.000) in the center of a white screen. After a variable and unpredictable interstimulus interval (ISI) ranging from 1,000 to 10,000 ms, in 1,000-ms increments, the zeros began counting upward. [Note this is a slight deviation from the design of Maybrier et al. (2023) but was based on other online administrations of the PVT to ensure more reliable timing and consistency in ISIs across participants; Welhaf and Kane, 2024]. Participants were instructed to stop the timer by pressing the space bar as quickly as they could once they noticed the numbers were counting upward. If participants did not respond within 9 s, that trial was counted as an error and removed. Once participants responded, the numbers stopped and were displayed for 500 ms to provide RT feedback. There were five practice trials to familiarize participants with the task. Participants then completed a total of 90 trials (nine at each ISI, which were randomly presented for each participant). The FP distribution was uniform for all participants.

Prior to beginning the PVT, participants were asked to put away any devices that may have been distracting (e.g., phone, e-mail, video games). Participants also answered several questions about their experiences during the study to screen out potential “bots,” or for participants who not fully engaged in the study either due to distracting environments or by engaging in media-multitasking behaviors during the study [adapted from Welhaf and Kane (2024)]. No participants failed the bot check or reported extreme levels of distraction, sleepiness, or media multitask during the study.

## Results

I followed an identical trial cleaning procedure as described in Study 1. First, I removed RTs to false alarms (i.e., hitting the spacebar during the ISI) and RTs below 200 ms. Finally, RTs greater than 3,000 ms were removed. This affected, on average, 2.66% of RTs across all participants. Consistent with previous research, I removed two participants who had an excessive number of missing trials (>10; Robison et al., 2023; Welhaf et al., 2025). Thus, the final sample consisted of 158 participants. Specifically, there were 78 younger adults (30 female, 46 Male, 2 Non-Binary) who were, on average, 27.74 years old (SD = 4.42 years) and 80 older adults (35 female, 44 Male, 1 Non-Binary) who were, on average, 69.93 years old (SD = 3.78 years).

### Effects of age on temporal preparation

**FIGURE 2:**
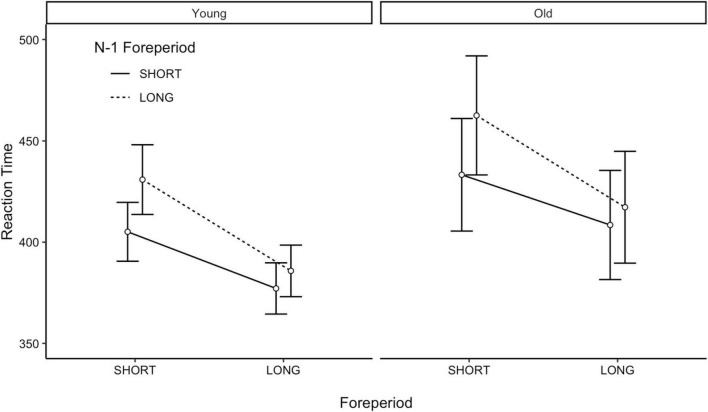
FP effects for each age group. Dots represent the condition mean and error bars are the 95% confidence intervals. FP, foreperiod.

The results of the linear mixed effect model are visualized in Figure 2. First, there was a just non-significant effect of age on RT, *b* = −31.68 (SE = 16.03), *p* = 0.050. Younger adults (*M* = 399, SD = 68) were significantly faster to respond during the PVT compared to older adults (*M* = 430, SD = 129).

As seen in Figure 2, there was a significant variable FP effect, *b* = 45.20 (SE = 3.77), *p* < 0.001. Across all participants, there was a significant slowing in responses when the current trial was a short FP duration (*M* = 433, SD = 107) compared to when the current trial was a long FP duration (*M* = 397, SD = 98). Additionally, the sequential FP effect was significant, *b* = −9.00 (SE = 3.80), *p* = 0.018. Across all participants, there was a significant slowing in responses when the previous trial was a long FP duration (*M* = 424, SD = 107) compared to when the previous trial was a short FP duration (*M* = 406, SD = 100). There was also evidence of the asymmetry in the sequential FP effect, *b* = −20.66 (SE = −3.85), *p* < 0.001. Critically, however, none of the FP-related effects were moderated by age (*p*’s > 0.678). Thus, despite being slower to respond, older adults demonstrated comparable temporal preparation abilities to younger adults.

### Study 2 discussion

Study 2 aimed to replicate the initial findings presented in Study 1 in an independent sample. Consistent with Study 1, and previous work using the PVT, I found evidence for both the variable and sequential FP effect in the PVT. Also consistent with Study 1 and previous literature, this effect was comparable across both younger and older adults. Thus, I appeared to replicate the key null effects reported in Study 1 that age did not moderate either the variable or sequential FP effects.

## General discussion

The present study presented a test of age differences in implicit timing abilities using a simple RT task designed to create a variable FP paradigm. The results of the current studies clearly demonstrated that while the variable (and to a lesser degree) sequential FP effects were robust, they did not differ in magnitude across the age groups. These findings replicate several previous findings that older adults have a largely comparable ability to prepare for action when not explicitly instructed to attend to time.

Given that across both studies, older adults showed a similar magnitude of the variable FP effect to younger adults, these data suggest that older adults do not rely more heavily on the hazard function than younger adults as suggested by others (e.g., Droit-Volet et al., 2019; Otsuka et al., 2023). While consistent with much of the literature on the variable FP effect in aging, this finding is perhaps somewhat surprising given recent work showing that younger adults with lower working memory capacity, who should have similar cognitive capacities as healthy older adults, showed a larger variable FP effect in a similar PVT task as the one used in the current study (Welhaf, 2024). This suggests that implicit timing ability appears intact at least among a seemingly healthy group of older adult participants.

One consideration for these null effects may be due to the methodological design of the PVTs in the current study and how they differ from traditional variable FP designs. Specifically, the PVT presented a larger range of FP options compared to standard variable FP studies. For example, Capizzi et al. (2022) included FP durations ranging from 480 to 1920 ms. The current design, however, ranged from 1 to 10 s. It is well-established that younger and older adults differ in their strategies to respond during speeded tasks with younger adults typically favoring speed and older adults favoring accuracy. As such, in shorter timing contexts, like previous studies, younger adults may be at an advantage. However, in the PVT, there is no true accuracy component and so strategy choice is less likely at play.

Rather, extending the possible FP distribution in the PVT likely taxed other cognitive processes. While younger and older adults differ in their basic attention control ability, there is some evidence to suggest that there is little difference between these groups in their ability to sustain attention over time (e.g., Vallesi et al., 2021). Thus, having these longer FP durations may have allowed older adults to work within a more favorable timeframe which could have reduced age difference in the specific modulation of the variable and sequential FP effects. The PVT is clearly a more vigilance demanding task while other simple RT tasks (with shorter FP durations) likely do not tax vigilance. Previous work has shown that younger are prone to lapses of attention or mind wandering, especially at longer intervals (e.g., Massar et al., 2020; Unsworth and Robison, 2020) which is less prevalent in older adults. Thus, the hypothesized age-related difference in the FP effect may have been difficult to observe due to other cognitive processes taking over.

Recent work has demonstrated that implicit timing might be disrupted in older adult populations that have clinical impairments including cognitive decline (Capizzi et al., 2022; but see Visalli et al., 2025 for further explanation), Mild Cognitive Impairment and Alzheimer’s Disease (Sylvain-Roy et al., 2010), and Parkinson’s Disease (e.g., Jurkowski et al., 2005; Mioni et al., 2018). Indeed, it has been previously argued that temporal preparation is an attentionally demanding process (e.g., Capizzi et al., 2012; Sylvain-Roy et al., 2010) and so deficits in implicit timing, and specifically, the variable FP effect, might only arise for groups that lack sufficient attentional abilities. Consistent with this notion, Droit-Volet et al. (2019) found no correlation between neuropsychological measures of memory and attention and performance in an implicit timing task. Future work will need to also consider the cognitive and neuropsychological profiles of older adults to confirm such a hypothesis.

Regarding the age-related differences in the sequential FP effect, younger and older adults showed similar levels of the more automatically driven temporal preparation process. This finding is consistent with the previous studies which reported no age differences in the sequential FP effect (e.g., Chauvin et al., 2016; Vallesi et al., 2009). Given the consistencies across studies, it appears likely that age differences in the bottom-up, and more automatic, aspect of temporal processing ability also remain intact across age groups. If this sequential FP effect represents basic arousal or alertness levels (e.g., Steinborn and Langner, 2012), it is possible then, that what these findings are reflecting are similarities in younger and older adult’s arousal levels. One way to address this more directly is to use physiological approaches such as pupillometry in the context of a variable FP paradigm (see Yamashita et al., 2022).

The design of the current studies may also have implications for addressing sequential FP effects. In more traditional variable FP studies, the limited range of FP possibilities, sequential effects become point-specific and the relevant probability of the trial occurring are clearly defined. For example, in a paradigm with FP durations of 500, 1,000, and 1,500 ms, the probability of a trial occurring increases as time goes by in a clear manner (i.e., once the 500 ms interval passes, there is a 50% chance of the 1,000 ms trial occurring; and, once both the 500 and 1,000 ms intervals occur, there is a 100% chance of the 1,500 ms interval occurring).

In the PVT, because the FP distribution is more continuous in nature, participants may create their own representations of what is deemed “short” vs. “long” on every trial. This may have reduced how easily sequential FP effects could be detected. While “distributional effects” have been known to influence the sequential FP effect (e.g., Han and Proctor, 2022; Los and Agter, 2005), uniform FP distributions appear to increase the magnitude of the sequential FP effect and its asymmetry (Los et al., 2001; Steinborn et al., 2009, 2010). Future studies should consider how the range and shape of the FP distribution contribute to age-related changes in temporal preparation ability.

### Alternative explanations and limitations

One possible explanation for the lack of age-related differences in these implicit timing abilities could also be related to motivational differences. Specifically, older adults commonly have higher levels of motivation to perform well on basic cognitive tasks (see Carr et al., 2022 for commentary on this matter). Indeed, previous research has shown that motivations aimed at altering motivation levels such as “try-harder” instructions (Steinborn et al., 2017) or rewards (Los and van den Heuvel, 2001; Massar et al., 2018) can alter implicit temporal preparation abilities, at least among younger adults. Thus, if older adults are, on average, more motivated, this might result in similar FP effects to those of younger adults. Future work will need to consider accounting for levels of motivation to address this potential confound.

Additionally, as alluded to earlier, the distribution of the FP may have large and unintended consequences for examining age-related differences in FP effects. While similar approaches have been used previously (e.g., Massar et al., 2020; Welhaf, 2024), it is also possible that grouping the FPs into short and long FPs with only a relatively few trials at each ISI may have limited statistical power to detect age-differences in the critical FP effects. Given the consistencies in the main findings across the two studies, I find this to be unlikely. Recent simulation work has demonstrated that even with smaller numbers of trials, larger sample sizes can overcome concerns of reduced statistical power (e.g., Miller, 2024 for similar arguments in studies of reaction time). With that in mind, future studies assessing age-related differences in FP effects may want to explicitly investigate how possible FP distribution might modulate the variable and sequential FP effect across younger and older adults to rule out such methodological concerns.

## Conclusion

Across two independent studies, I used a novel approach to assess age-differences in the variable and sequential FP effect in a simple RT task. The present studies provide consistent evidence that implicit timing abilities are largely preserved in healthy aging. Specifically, despite being slower overall, older adults showed similar variable and sequential FP effects to those of younger adults. Future work will need to address how this lack of age-differences fit into existing theories of temporal preparation ability.

## Data Availability

The datasets presented in this study can be found in online repositories. The names of the repository/repositories and accession number(s) can be found below: https://osf.io/zmqxf/overview?view_only=77ba6b8c41674d9f849de22d05f0cb53.

## Footnotes

1
https://osf.io/zmqxf/overview?view_only=77ba6b8c41674d9f849de22 d05f0cb53

2
https://www.prolific.co

3
https://gorilla.sc
